# Pea Marker Database (PMD) – A new online database combining known pea (*Pisum sativum* L.) gene-based markers

**DOI:** 10.1371/journal.pone.0186713

**Published:** 2017-10-26

**Authors:** Olga A. Kulaeva, Aleksandr I. Zhernakov, Alexey M. Afonin, Sergei S. Boikov, Anton S. Sulima, Igor A. Tikhonovich, Vladimir A. Zhukov

**Affiliations:** 1 All-Russia Research Institute for Agricultural Microbiology, Podbelsky chausse, Saint-Petersburg, Russia; 2 Saint-Petersburg State University, Universitetskaya embankment, Saint-Petersburg, Russia; National Bureau of Plant Genetic Resources, Pusa India, INDIA

## Abstract

Pea (*Pisum sativum* L.) is the oldest model object of plant genetics and one of the most agriculturally important legumes in the world. Since the pea genome has not been sequenced yet, identification of genes responsible for mutant phenotypes or desirable agricultural traits is usually performed via genetic mapping followed by candidate gene search. Such mapping is best carried out using gene-based molecular markers, as it opens the possibility for exploiting genome synteny between pea and its close relative *Medicago truncatula* Gaertn., possessing sequenced and annotated genome. In the last 5 years, a large number of pea gene-based molecular markers have been designed and mapped owing to the rapid evolution of “next-generation sequencing” technologies. However, the access to the complete set of markers designed worldwide is limited because the data are not uniformed and therefore hard to use. The Pea Marker Database was designed to combine the information about pea markers in a form of user-friendly and practical online tool. Version 1 (PMD1) comprises information about 2484 genic markers, including their locations in linkage groups, the sequences of corresponding pea transcripts and the names of related genes in *M*. *truncatula*. Version 2 (PMD2) is an updated version comprising 15944 pea markers in the same format with several advanced features. To test the performance of the PMD, fine mapping of pea symbiotic genes *Sym13* and *Sym27* in linkage groups VII and V, respectively, was carried out. The results of mapping allowed us to propose the *Sen1* gene (a homologue of *SEN1* gene of *Lotus japonicus* (Regel) K. Larsen) as the best candidate gene for *Sym13*, and to narrow the list of possible candidate genes for *Sym27* to ten, thus proving PMD to be useful for pea gene mapping and cloning. All information contained in PMD1 and PMD2 is available at www.peamarker.arriam.ru.

## Introduction

Modern plant breeding relies on selection of genotypes with desirable traits by means of marker-assisted selection (MAS) and genomics-assisted breeding (GAB) [1,2]. Additionally, close attention is paid to identifying genes of interest by the candidate gene approach [3]. In cereals, significant success in both MAS and GAB has already been achieved, but in pulse crops the implementation of these approaches has been limited, mostly due to scarceness of available genomic resources and lack of optimized bioinformatics tools [2,3].

Garden pea (*Pisum sativum* L.) is one of the most valuable pulse crops, an integral part of agricultural systems throughout the world, and a model object in plant genetics since the days of Gregor Mendel [4,5]. However, modern pea genetics is lagging behind that of model plants, despite the significant progress made in discovery of symbiotic genes and the presence of available collections of unique mutant lines, for example, impaired in nitrogen-fixing symbiosis and arbuscular mycorrhiza development [6]. Pea has a large genome (1C = 4300 Mb) [7] congested with repetitive elements [8] complicating genome assembly; therefore, transcriptome assemblies of different tissues and organs are the most comprehensive source of pea gene sequences available [9–12]. Although the transcriptome assemblies alone are not very useful in studies concerning search for particular pea genes, they can provide the basis for construction of high-density genetic maps, which are the crucial tools for identification of loci and markers associated with traits of interest.

The first example of genetic linkage in pea was described in 1912 [13], and the first genetic map was constructed in 1925 [14], but only in the early 2000s complete genetic maps composed of 7 linkage groups (LGs) consistent with the pea karyotype were constructed mostly based on “anonymous” RFLP and RAPD markers [15–18]. Later, with the development of sequencing technologies and the emergence of pea EST databases, the first maps based on genic markers were built [19,20]. High level of genome synteny between *P*. *sativum* and *Medicago truncatula* Gaertn., a model legume with sequenced and annotated genome, opens the opportunity of comparative analysis between pea linkage groups and chromosomes of *M*. *truncatula*. This approach makes it possible to determine the nucleotide sequence of specific pea genes through fine gene mapping and subsequent search for candidate genes in *M*. *truncatula* and, possibly, other related species with sequenced and annotated genomes [5,21].

In recent years, numerous gene-based molecular markers have been designed and mapped in *P*. *sativum*. Development of “next-generation sequencing” (NGS) technologies allowed identifying thousands of single nucleotide polymorphism sites (SNPs) across a species’ genome, as demonstrated by several works aimed at polymorphism studies and construction of pea genetic maps [22–30]. These works are based either on transcriptome sequencing [22,24–28], or on alternative technologies such as RADSeq (Restriction site Associated DNA Sequencing) method (both reducing genome complexity), or whole genome sequencing technology [23,29,30]. However, the majority of genome-based markers are associated with random, often non-coding parts of genome rather than with particular functional transcripts, thus denying researchers the possibility of exploiting the synteny with *M*. *truncatula* or other legumes for candidate gene search.

Although a number of online marker databases exists, none of them provide information accumulated from different sources in a simple and convenient format [31,32], while support for others has been dropped entirely [33]. The need to gather information from different papers hinders the work on locus localization within a LG and further searches for candidate genes. Thus, the aim of this work was to integrate the information about pea markers and provide an easy-to-use online tool–the Pea Marker Database (PMD)–combining information about known pea gene-based markers.

At first, PMD1 was constructed to combine the markers from several independent sources [19,24–26,34–37]. By the time the development of PMD1 was finished, a highly comprehensive genetic map based on analysis of 12 pea mapping populations and consisting of new SNP markers and previously obtained markers from different studies [15,17,19,20,24–26,34–36,38–51] was constructed [27]. Unfortunately, the resultant marker positions were presented in large data files as supplementary material, and were, in our opinion, not convenient for intensive marker analysis. Thus, we transformed the data obtained by Tayeh and colleagues in 2015 [27] to a web-based, user-friendly form with additional important features, which we called PMD2.

Here, we present the description of the databases and the related online interface, as well as demonstrate the databases usability by (i) marker design for the particular pea genome region, followed by (ii) fine mapping of pea symbiotic genes *Sym13* and *Sym27*, and (iii) subsequent candidate gene search.

## Materials and methods

### Data sources

Markers for PMD1 were obtained from eight papers [19,24–26,34–36,38] and divided into four groups, or sets. Markers designed using NGS data were labeled according to the name of the first author of the corresponding article: **Dm** set (Duarte marker set) [24], **Sm** set (Sindhu marker set) [25] and **Lm** set (Leonforte marker set) [26]. The sequences of the markers in these sets were obtained from the supplementary files of the corresponding articles [24–26]. The fourth **ESTm** set (EST marker set) included EST markers [19,34–36,38] used to construct the previous pea genetic maps. The sequences of the EST markers were obtained from the NCBI nucleotide database [52].

The whole set of markers for PMD2 development was obtained from [27]. Of those, markers based on the transcripts from the work of Alves-Carvalho and colleagues in 2015 [12] were labeled **ACm** (Alves-Carvalho marker set) and used for PMD2 development along with **Dm**, **Sm** and **ESTm** sets, as well as with several markers from a range of previous works [15,17,20,39–51,53] not combined into a set (“separate markers”). The sequences for the markers in the **ACm** set were obtained from the pea transcriptome assembly available online at http://bios.dijon.inra.fr/FATAL/cgi/pscam.cgi [54]. The sequences of the nucleotide-based separate markers [20,39–48,53] were obtained from the NCBI database.

### PMD1 construction

As the first step of PMD1 construction, BLASTN analysis against the *M*. *truncatula* genome (Mt 4.0 v.1) [37] with a threshold E-value of 10^−10^ was conducted for all sequences from the **Dm**, **Sm** and **Lm** sets. This revealed a number of duplicated (i.e. corresponding to the same *M*. *truncatula* gene) markers in each set. The **Dm** set had the best combination of the overall number and distribution of markers and the minimal number of identified duplicated markers, which made it the most suitable “backbone” for artificial map construction.

In total, highly similar *M*. *truncatula* gene sequences were found for 96.7% of the pea marker genes. Genomic synteny analysis showed that the pea LGs I, II, III, V, and VI corresponded to the *M*. *truncatula* chromosomes 5, 1, 2/3, 7, and 2/6, respectively, consistent with previously reported results [5,20]; pea LG IV corresponded to regions of chromosomes 8, 5 and 4, and LG VII–to regions of chromosomes 8 and 4 of *M*. *truncatula*.

Further analysis showed that LGs from different studies were inverted relative to each other. For example, the order of the markers in the LGs I, III and VII in the **Lm** map was inverted relative to the corresponding LGs in the **Dm** and **Sm** maps; the marker order in the LGs IV and VI in the **Sm** map was also inverted relative to the corresponding LGs in the **Dm** and **Lm** maps. In order to construct a comprehensive database, LGs from all maps were oriented uniformly.

Markers from different datasets corresponding to the same transcript of *M*. *truncatula* were deemed “identical” and used as anchor markers for map joining. Since the locations of the markers in each set were determined using different mapping populations and, moreover, different joining algorithms for each map, the distances between the anchor markers varied in each set. To equalize map scales, the distances between markers belonging to the **Sm** and **Lm** sets were scaled to the dimension of the **Dm** set; then, markers lying between each pair of anchor markers were placed accordingly on the constructed “artificial” LGs.

**Fig 1:**
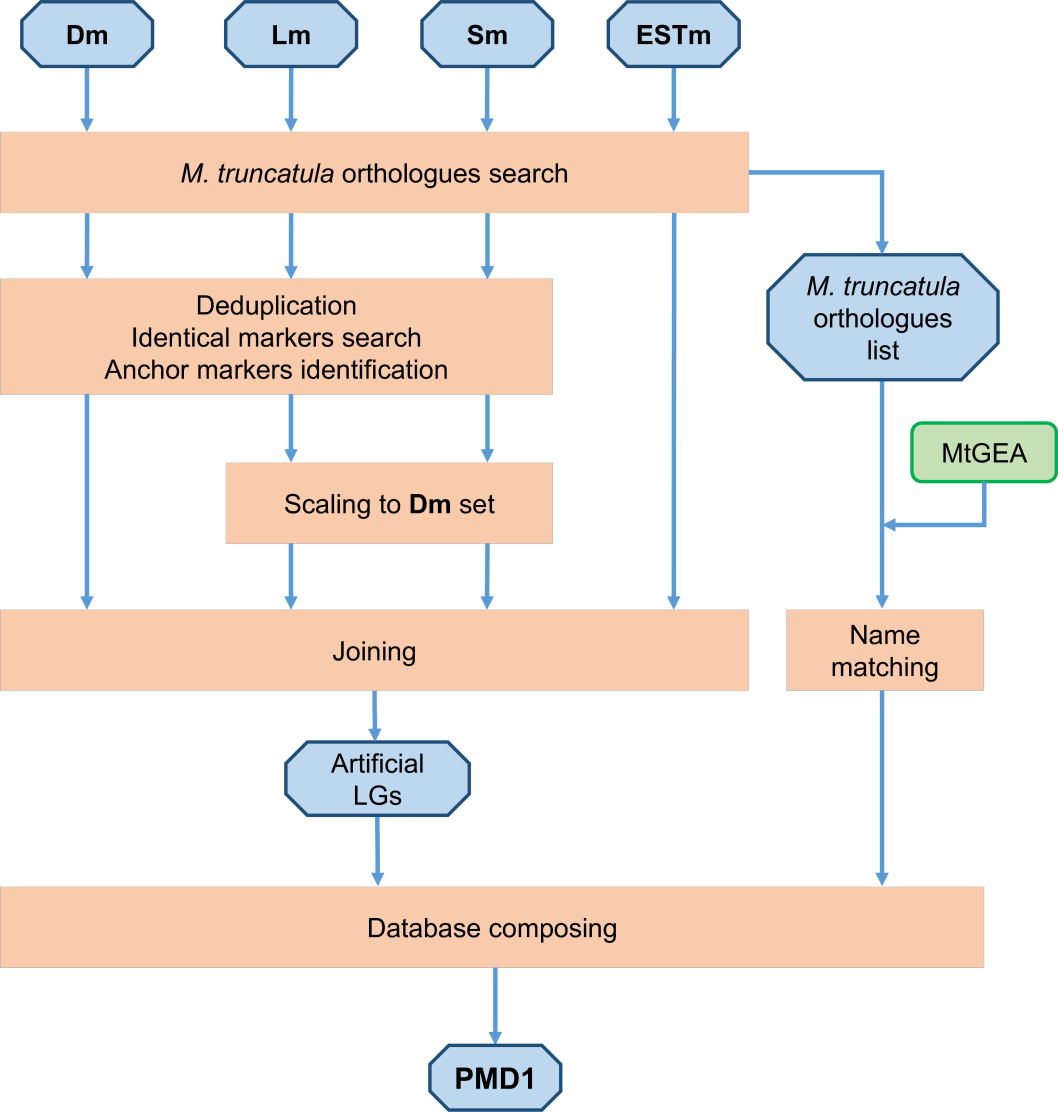
Pipeline of the PMD1 development. Initial and resulting datasets are placed in blue octagons. Operations are placed in peach-colored rectangles. Steps of PMD1 development are indicated by arrows. MtGEA means *Medicago truncatula* Gene Expression Atlas.

Finally, by aligning the sequences from the **ESTm** set to the *M*. *truncatula* genome, correspondence between the NGS-based and EST-based markers was identified. All identified marker names were included in the description of the corresponding loci on the resulting PMD1 map. This pipeline (Fig 1) combining the locations of markers from different studies resulted in a more comprehensive albeit potentially less accurate map; small discrepancies were found in the interposition of some markers which are most probably the result of combining maps from three different studies that varied in marker saturation in some regions and were based on different genetic lines (possibly carrying minor chromosome rearrangements).

Functional annotation of the markers included searching for homologous sequences in the *M*. *truncatula* genome and identifying the relevant entries (referred to as “Name matching” in Fig 1) in the *M*. *truncatula* Gene Expression Atlas (MtGEA) [55,56].

The artificial LGs construction was performed using an original program developed within the frame of this study in Visual Basic for Applications [57]. Visualization of the database content was conducted using D3.js, a JavaScript library for manipulating documents based on [58].

### PMD2 construction

**Fig 2:**
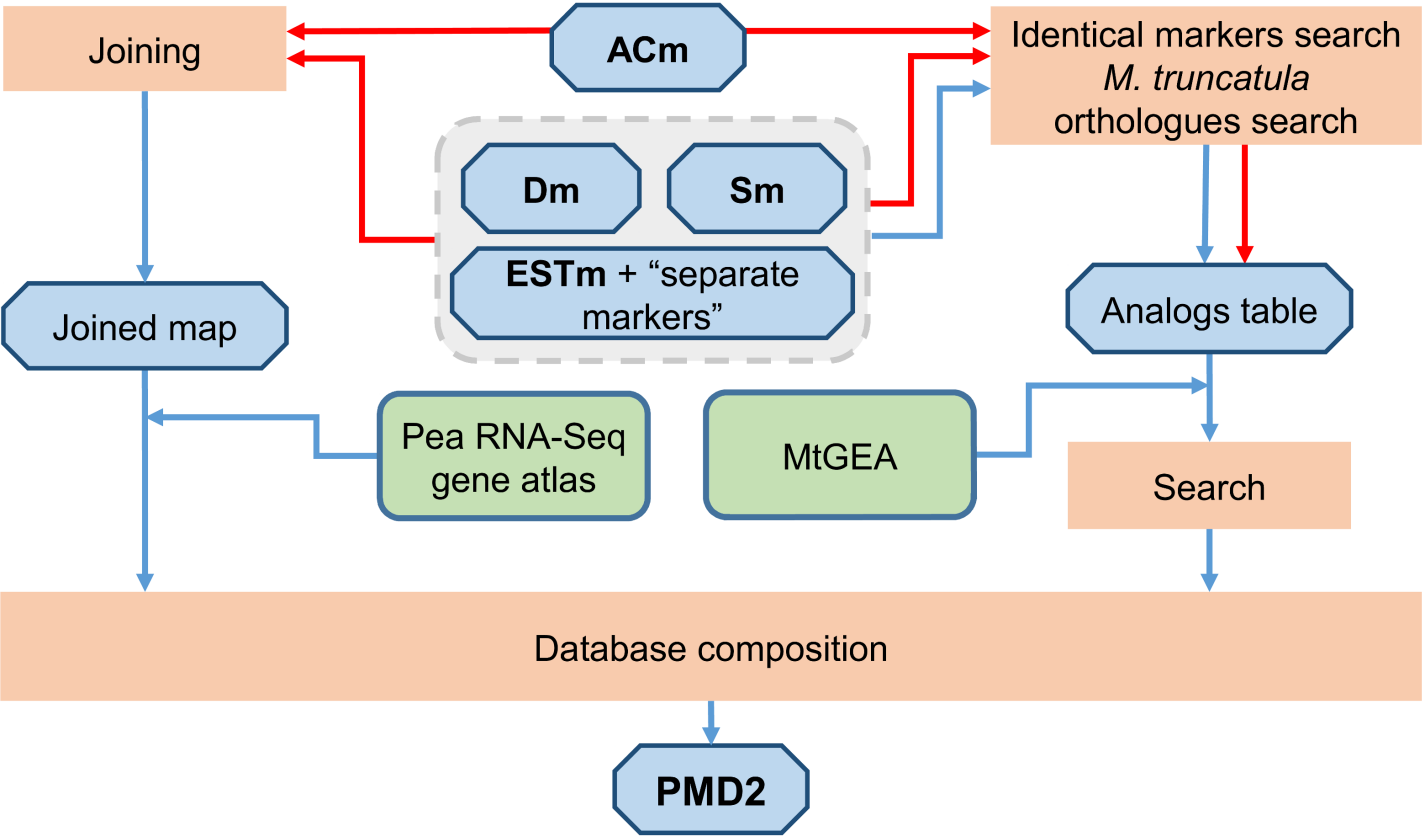
Pipeline of the PMD2 development. Initial and resulting datasets are placed in blue octagons. Operations are placed in peach-colored rectangles. Steps performed previously by Tayeh and colleagues in 2015 and described in [27] are indicated by red arrows. Steps performed in the course of the present study are indicated by blue arrows. MtGEA–*Medicago truncatula* Gene Expression Atlas.

For PMD2 construction, the markers positions, corresponding transcript names, references to previously developed markers [15,17,19,20,24,25,34–36,38–51], *M*. *truncatula* homologous sequences for the **ACm** set and information concerning the quality of some markers were taken from the supplementary material data files of Tayeh and colleagues [27] and processed (Fig 2).

As gene and QTL mapping studies in pea had been conducted by different research groups using independently developed markers [19,20,24,25,34–36,38–48,53], the correspondence between these markers and the **ACm** set required examination. Table of correspondence between markers from the **ACm**, **Dm, Sm** sets and markers from [20] was extracted from the supplementary material data files of [27]. Markers found to be developed from the same sequence were labeled “identical” (as in PMD1). Comparative analysis of the markers showed a high number of duplications in [26] with up to 6 distinct markers corresponding to a single marker from [27] (consistent with the analysis carried out for markers from **Lm** set during PMD1 development). To avoid complicating the database data from [26] was not included in PMD2.

For marker sets from [19,34–36,38–48,53], comparative analysis between marker reference sequences (EST or nucleotide) and transcript sequences from the **ACm** set was performed using the BLASTN algorithm (construction of “Analogs table” in Fig 2). Markers with a single hit with more than 95% coverage were marked as “identical” (3411 markers in total corresponding to 1586 distinct transcripts in PMD2).

The similarity between marker sequences [19,20,24,25,34–36,38–48,53] and *M*. *truncatula* sequences was assessed using the BLASTN algorithm (construction of “Analogs table” in Fig 2). Based on the results of this analysis, correspondence to the relevant *M*. *truncatula* genes was found for 75.8% of the investigated sequences. Functional annotation of the markers also included identifying the relevant entries in the *M*. *truncatula* Gene Expression Atlas (MtGEA) [55,56]. The correspondence between all genic markers from PMD2 and the expression profiles of the corresponding *M*. *truncatula* transcripts available in MtGEA was found for 61% of the sequences.

The functional annotation of markers from the **ACm** set includes information about the expression profile of the corresponding transcript from Pea RNA-seq Atlas available online at http://bios.dijon.inra.fr/FATAL/cgi/pscam.cgi [12].

Visualization of the database content was conducted using D3.js, a JavaScript library for manipulating documents based on [58]. The databases have been tested on Mozilla Firefox (version 49.0.1) and Google Chrome (version 53.0.2785.143) browsers.

### Plant growth conditions and gene mapping

The pea mutant line E135F, obtained after ethyl methanesulfonate (EMS) treatment of cv. Sparkle seeds [59] and carrying the recessive allele of *sym13* gene [60] corresponding to manifestation of the mutant phenotype (white nodules not capable of nitrogen fixation, so-called Fix^-^ phenotype), was crossed with the multiply marked line JI73 (formerly NGB1238) characterized by normal nodulation. After self-pollination of the resultant F_1_ plants, the mapping population was obtained. For analysis of the symbiotic phenotype, F_2_ plants were grown in 5 L plastic pots containing quartz sand with mineral nutrition lacking combined nitrogen [61] and inoculated with *Rhizobium leguminosarum* bv. *viciae* strain RCAM 1026 [62]. After 28 days of growth, the plants were taken from the pots and the phenotype of root system was examined. In total, 72 plants were analyzed (18 Fix^-^ and 54 Fix^+^, Chi-square for 1:3 = 0.00, p-value = 1.00).

The pea mutant line RisFixQ obtained after EMS mutagenesis of cv. Finale seeds [63] carries the recessive allele of *sym27* gene causing formation of non-fixing, prematurely senescent nodules [64]. The *Sym27* mapping population consisted of 50 F_2_ plants obtained by crossing of the RisFixQ (*sym27*) line and the multiply marked line JI73 (NGB1238), followed by self-pollination of F_1_ plants. The symbiotic phenotypes of F_2_ plants were assessed by analyzing F_3_ families originating from each F_2_ plant (10 plants from each F_3_ family were planted per one 5 L plastic pot and grown in the same conditions as for *Sym13* mapping experiment). At the 28^th^ day after planting and inoculation, the root system phenotype was examined; families containing only plants with normal nodules were scored as descendants of *Sym27* F_2_ plants, families containing only plants with defective nodules were scored as descendants of *sym27* F_2_ plants, and families containing plants with either normal or defective nodules were scored as descendants of F_2_ plants heterozygous for *Sym27*). Thus, the allelic state of *Sym27* was analyzed as a co-dominant marker (12 +/+, 24 +/-, 11 -/-, Chi-square for 1:2:1 = 0,06; p-value = 0,97).

DNA was extracted from the plant leaves according to [65] with slight modifications. RNA was extracted from nodules of pea mutant line E135F collected on the 21^st^ day after inoculation. The growth conditions of plants used for cDNA analysis and protocols of RNA extraction and cDNA synthesis were described previously [66,67]. PCR was performed in iCycler (Bio-Rad, USA) or Dyad (Bio-Rad, USA) with use of the ScreenMix-HS kit (Evrogen, Russia) in the conditions as follows: 95°C (5 min), 35 x [95°C (30 sec), 58–60°C (various for different primer pairs) (30 sec), 72°C (1 min)], 72°C 5 min. Primer design was performed with help of the Primer-BLAST [68]. For CAPS marker analysis, the proper restriction enzyme for SNP site recognition was chosen using dCAPSFinder 2.0 (http://helix.wustl.edu/dcaps/dcaps.html) [69], or was extracted from the list of available transcriptome-based pea markers for the lines Sparkle (parent of E135F (*sym13*)), Finale (parent of RisFixQ (*sym27*)) and JI73 (= NGB1238) [28]. Restriction digest of the PCR product was performed with use of Fermentas enzymes (Thermo Fischer Scientific, USA). Information regarding PCR primers and restriction enzymes is listed in S1 and S2 Tables. Primers for amplification of the candidate gene *Sen1* were designed on the basis of transcript GDTM01047803 found in pea nodule transcriptome assembly [11]: PsSen1_fw1: 5’-TAAACAGATCAATCAAGCATTCATG-3’, and PsSen1_rv1: 5’-ATTGGTTCAACATGAAGTATACG-3’.

The genetic linkage maps were constructed using JoinMap 4.1 software with default parameters [70]. For visualization of the maps, the MapChart program [71] was used.

The sequence of pea *Sen1* determined for cv. Sparkle is deposited in GenBank under the accession number KY888171.

The molecular biology procedures were performed using equipment of the Core Center “Genomic Technologies, Proteomics and Cell Biology” in ARRIAM, Saint-Petersburg, Russia.

## Results and discussion

### Database description

PMD resource was developed to provide information about pea gene-specific markers in a simple and convenient format. Both databases allow selecting linkage groups and markers along with searching for specific markers with the “search” function. For each LG, a specific region can be quickly selected and the scale can be adjusted. Specific marker can be selected by clicking on the square adjacent to the marker. Marker selection evokes a table containing information about the name of the marker, LG, literature references and the sequence of the transcript. Additionally, the table provides information about the homologous sequence from *M*. *truncatula* with reference to the dedicated transcript page in the Phytozome resource [72], and information about the expression profile of the *M*. *truncatula* transcripts in the MtGEA [56]. Note that for transcripts corresponding to a large number of entries in MtGEA, PMD returns the first record.

**Fig 3:**
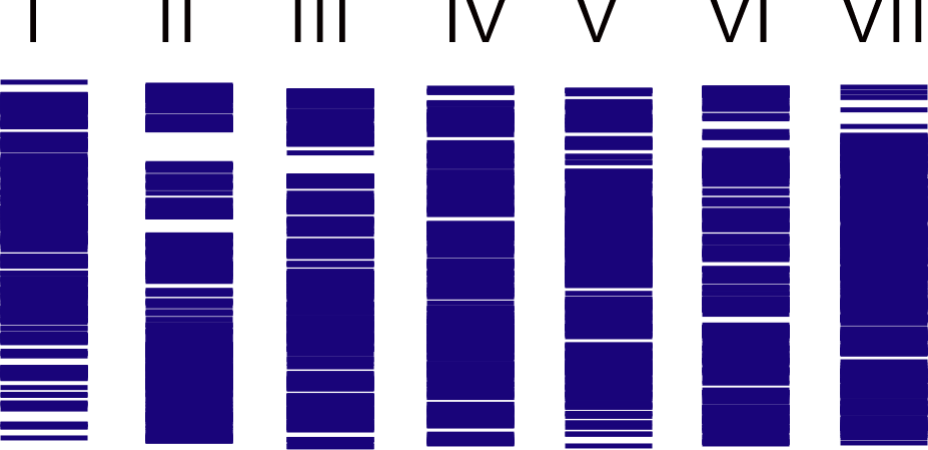
Marker distribution across the artificial LGs presented in PMD1.

In total, PMD1 comprised 2484 markers: 336 in LG I, 328 in LG II, 404 in LG III, 317 in LG IV, 331 in LG V, 304 in LG VI and 464 in LG VII. The marker distribution across the artificial LGs is shown in Fig 3. For two or more markers representing the same pea transcript, all marker names were assigned to one locus in PMD. The average distance between markers was 0.6 cM 98.8% of the markers were spaced less than 4 cM apart. Most of the discrepancies resulted from map joining (see Materials and Methods; PMD1 development) are not exceed 3 cM and therefore are not critical for primary mapping. Nevertheless, markers with a dubious location are colored red in PMD1 and should be used cautiously in fine mapping studies.

**Fig 4:**
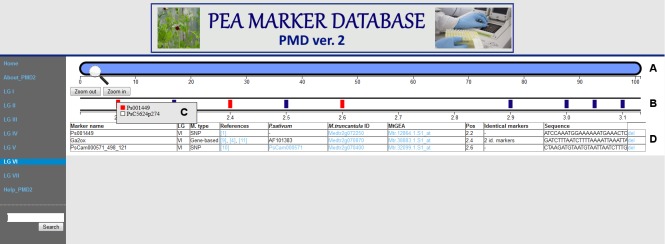
Interface of PMD2. (A) General view of selected LG. (B) Scaled region of selected LG. Previously selected loci are colored in red. (C) Markers situated in selected locus. (D) Table containing information about selected markers.

The later version, PMD2, contains a total of 15944 pea markers and has a few advanced features compared to PMD1. The linkage groups in PMD2 are arranged horizontally (Fig 4) for better representation of markers information. In PMD2, an advanced “search” function is implemented making it possible to search not only for a marker name (even incomplete), but also for a *M*. *truncatula* gene identifier. With *M*. *truncatula* gene identifier as a query the search is performed for a complete match. For identifiers not found in the database, the identifier of a nearest (calculated using *M*. *truncatula* genome map) gene present in PMD2 is shown in the output, a useful feature for researchers possessing only the information about the homology of investigated gene to *M*. *truncatula* genes.

PMD2 includes the information about “identical” markers and marker type (gene-based, AFLP, SSR, morphological, protein, RAPD, SNP). For some markers which were marked as “identical”, but had not been included in joined LGs in [27], only information about “identity” and literature reference is presented in PMD2. According to Tayeh and colleagues [27], some markers displayed a high level of segregation distortion and, therefore, were labeled as “HLSD” in PMD2. Moreover, by searching for *M*. *truncatula* sequences homologous to pea transcripts from [12], several highly similar sequences not located in a conserved syntenic block were revealed [27]. Such possible “not real orthologues” of *M*. *truncatula* are marked as “NRO” in PMD2. Also, for some markers labeled as “identical’, different *M*. *truncatula* sequences were revealed as the closest homologues, perhaps due to minute differences in hit scores obtained in BLAST search. Such markers are marked as “DH” in PMD2.

All information contained in PMD1 and PMD2 is available at www.peamarker.arriam.ru. Detailed instructions on the use of PMD1 and PMD2 are presented in the “Help” section at www.peamarker.arriam.ru.

### Comparative analysis of the marker positions in PMD1 and PMD2

Marker placement in the artificial LGs developed for PMD1 was compared to that in LGs presented in [27]. The analysis was carried out only for markers present in both PMD1 and PMD2. For all artificial LGs, the average number of markers with altered positions did not exceed 5%. Most of these markers were placed at the same site, possibly shifted by one or two markers in comparison with markers from [27], due to the different mapping scales in different studies, which does not strongly affect mapping success.

Although LGs IV, V and VI in PMD2 were found to be inverted in comparison with PMD1, it was decided to retain the order of the PMD2 markers as in the original paper of Tayeh and colleagues [27].

### Validation and applications

#### Mapping of *Sym13* gene

**Fig 5:**
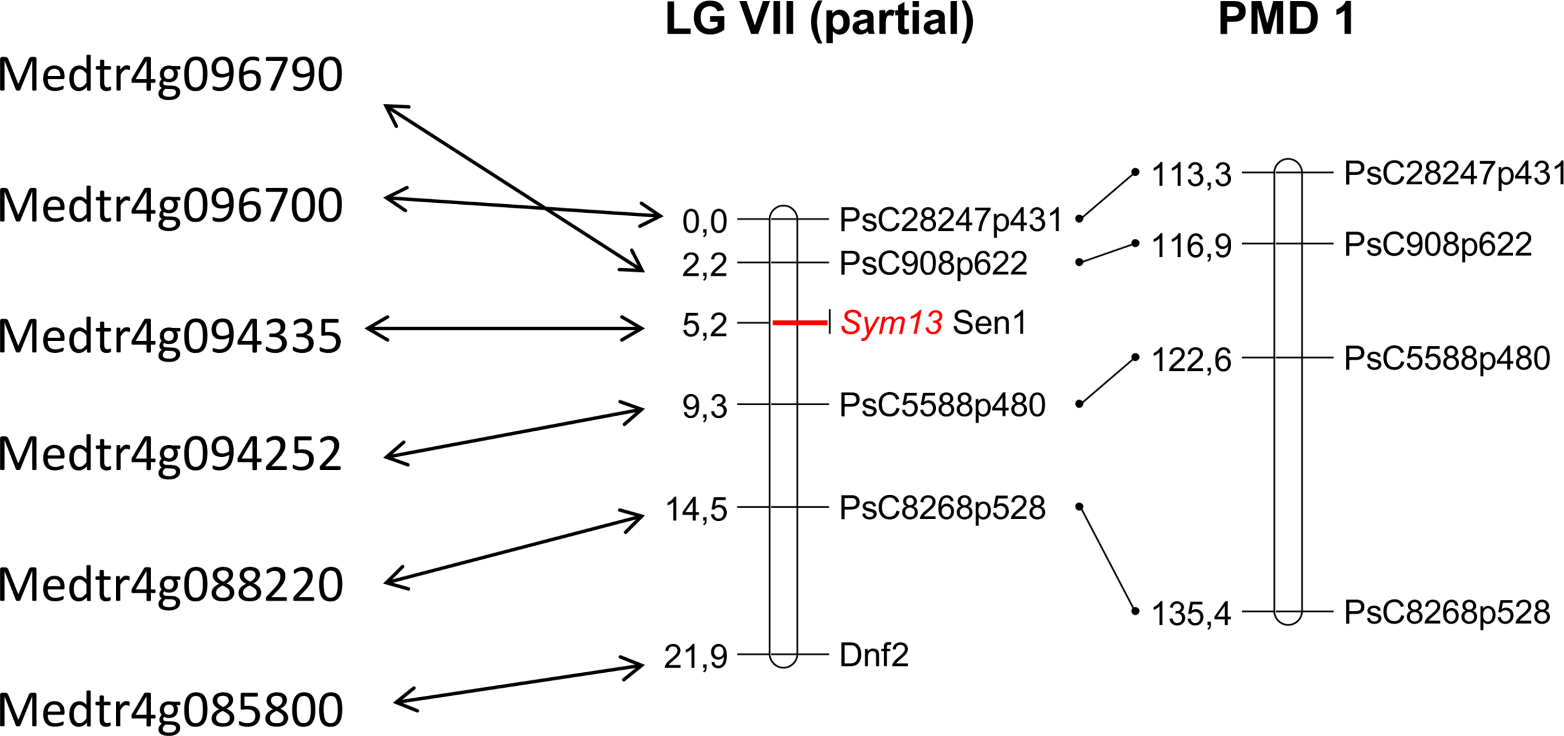
Localization of *Sym13* gene in relation to gene-based markers. From left to right: names of *M*. *truncatula* genes homologous to pea markers used for *Sym13* mapping, genetic map containing *Sym13*, artificial map from PMD1.

To test the usability of the PMD, we performed precise mapping of pea symbiotic gene *Sym13* using the gene-based markers developed in accordance to PMD1. Earlier, *Sym13* was shown to be genetically linked to allozyme markers *Skdh* and *Est-2* [60], which position is now considered as the middle of linkage group VII [20]. Therefore we (i) selected four markers from the middle part of LG VII, i.e. spanning the region from 110 cM to 140 cM according to PMD1 (S1 Table), (ii) investigated the polymorphism of the marker sequences using transcriptomic RNAseq datasets for Sparkle (parent of E135F (*sym13*)) and JI73 (= NGB1238) [28] (see Materials and Methods for details), (iii) found SNP sites suitable for CAPS marker design and made PCR primers flanking these sites (S1 Table). Segregation of the CAPS markers was then tested on F_2_ population, along with analysis of the gene-based marker designed for pea homologue of *M*. *truncatula DEFECTIVE IN NITROGEN FIXATION 2* (*DNF2*), which was initially chosen as a candidate for *Sym13* gene (S1 Table). As a result, pea *Sym13* was placed between genic markers *PsC5588p480* and *PsC908p622*, apart from *Dnf2* (Fig 5). The positions of all markers on the resulted map are in agreement with PMD1 markers order.

The successful localization of *Sym13* allowed us to carry out the search for candidate gene in *M*. *truncatula* genome. Since the markers flanking *Sym13* correspond to *Medtr4g096700* and *Medtr4g094252* genes, all genes in between were considered as possible candidates. Note that, as some recombination events were detected between *Dnf2* marker and *Sym13*, *DNF2* was excluded from the candidate genes list. Among the candidates, the gene *Medtr4g094335* attracted our attention as it was a homologue of known symbiotic gene *STATIONARY ENDOSYMBIONT NODULE 1* (*SEN1*) of *Lotus japonicus* (Regel.) K. Larsen [73], and, according to Pea RNA-seq Atlas (S3 Table), the expression of the corresponding pea gene was nodule-specific. We sequenced the pea *SEN1* homologue in E135F (*sym13*) and wild-type line Sparkle and found a nucleotide substitution c.C197T leading to amino acid change S66L in the case of mutant, which was predicted to be potentially damaging for protein function according to SIFT program [74]. Sequencing of the corresponding transcript on cDNA identified the same substitution, as well as the absence of introns, in pea gene *Sen1*, which is consistent with structure of *L*. *japonicus SEN1* gene and predicted structure of *M*.*truncatula* homologue *Medtr4g094335*. Thus, use of PMD for marker selection and candidate gene search in *M*. *truncatula* allowed us to infer that pea *Sym13* most likely encodes symbiosis-specific transporter of iron ions, as the presumable orthologous *SEN1* gene in *L*. *japonicus* does; however, mutant phenotype complementation studies are required to support the proposition about orthology of pea *Sym13* and *L*. *japonicus SEN1*.

#### Mapping of *Sym27* gene

As a second case study aimed at testing the features and facilities of PMD1 and PMD2, the pea symbiotic gene *Sym27* was fine-mapped in LG V based on the information provided in the database. Previously, the *Sym27* locus was roughly localized in LG V in relation to the gene-based markers *Pgd*, *Pme1* and *Met2* [53]. In the present study, we (i) identified *M*. *truncatula* genes homologous to the specified markers, (ii) found adjacent *M*. *truncatula* genes for which homologous pea markers were listed in PMD, (iii) outlined the region of LG V where, according to PMD, *Sym27* is located, (iv) selected markers lying 2–5 cM apart, and (v) adapted the selected markers for our mapping population (i.e., identified marker sequence variants between the parental lines of the mapping population and designed CAPS markers) and finally used them for *Sym27* fine mapping (S2 Table).

**Fig 6:**
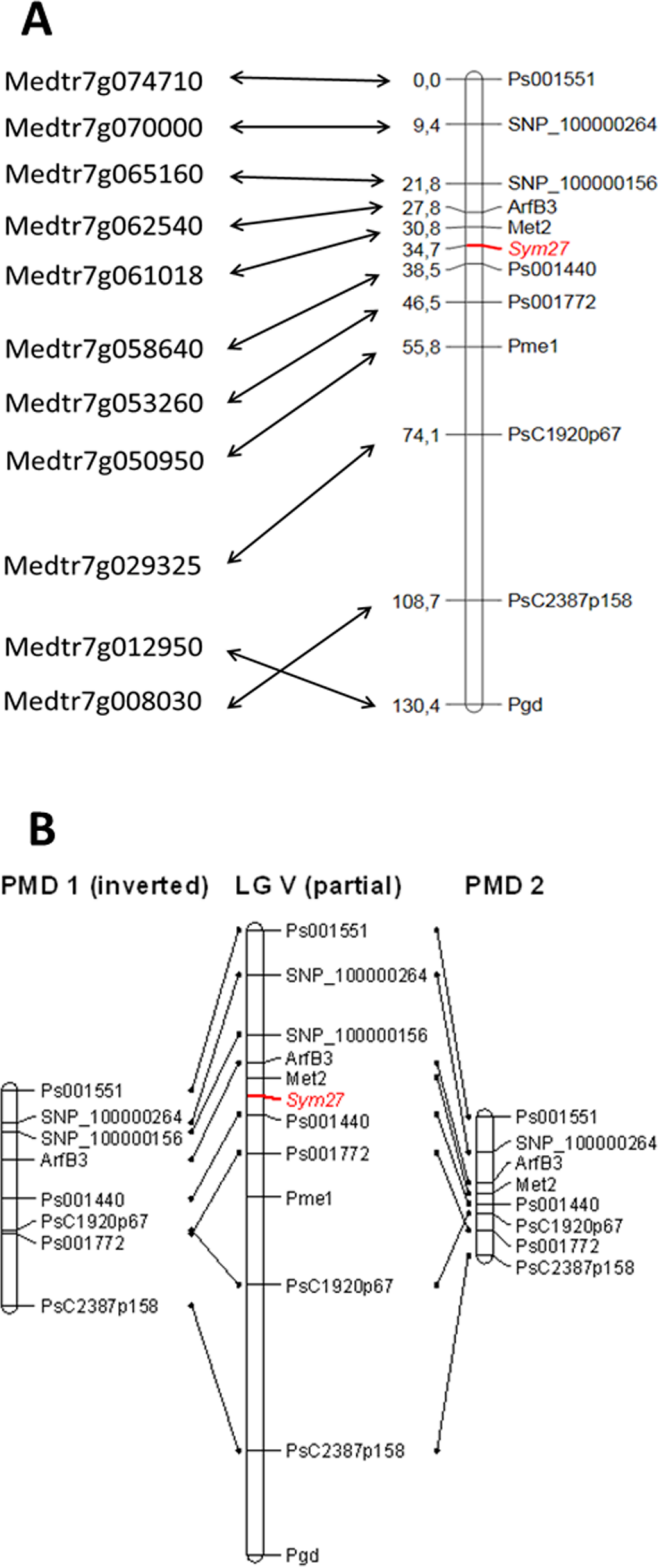
Localization of *Sym27* gene in relation to gene-based markers. A—*M*. *truncatula* genes homologous to pea markers used for *Sym27* mapping (right) and pea genetic map with *Sym27* position (left). B—Order of markers on the resulting map (center), PMD1 (left) and PMD2 (right).

As a result of mapping, the *Sym27* gene was localized in relation to nine gene-based markers between *Ps001440* and *Met2*, implying that the *Sym27* homologue in *M*. *truncatula* is located between the corresponding *Medtr7g058640* and *Medtr7g061018* genes (Fig 6). Note that, apart from LG V being inverted in PMD1 compared with PMD2, the order of markers on our genetic map was in good agreement with the “artificial” PMD1 and “real” PMD2 (Fig 6). According to the most recent *M*. *truncatula* genome release Mt4.0v1 [37], this region contains 121 genes, of which 10 can be considered promising candidates based on their nodule-specific expression, involvement in nitrogen assimilation or microbial hosting processes in the plant tissues (S4 Table). By contrast to *Sym13* candidate gene search, none of the *Sym27* candidate genes were previously reported as symbiotic, which complicates the analysis but pointing at the possible novelty of the *Sym27* gene function in nitrogen-fixing symbiosis.

## Conclusion

The increasing amount of genetics and genomics data obtained for model and agriculturally important plants provides a reliable basis for using up-to-date methods of molecular biology and genetics like MAS and GAB for crop breeding. Identification of particular genes responsible for desirable agricultural traits is usually performed via genetic mapping followed by candidate gene search, and such mapping is best carried out using gene-based molecular markers. Development of molecular markers leads to the construction of highly saturated genetic maps, which are essential tools for undertaking MAS and GAB. However, the usefulness of genetic and genomic data is highly dependent on availability of resources combining different sorts of such information.

This work was an attempt to integrate a large amount of information for pea gene-based markers into one database with a clear and user-friendly interface. Since the pea genome assembly is not available yet, comparative analysis between *P*. *sativum* and *M*. *truncatula* sequences is routinely used (for example, when searching for exon-intron junctions), making usability the defining and most important trait of any marker database. The Pea Marker Database (PMD) is a convenient tool that, as was demonstrated in the case study, facilitates marker development and gene mapping in pea. Indeed, combining the data on marker location that are collected in PMD with recently developed set of potential RNAseq-based markers [28] for some pea genotypes allowed us to identify the prominent candidate gene *Sen1* for pea symbiotic gene *Sym13* described ca. 30 years ago [60], a result important to further the understanding of the molecular mechanisms underlying nodule senescence in pea [66,67]. The fine mapping of the pea symbiotic gene *Sym27* based on the information provided in the PMD allowed us to narrow the list of possible candidate genes for *Sym27* to approximately 10. Thus, PMD will be useful for geneticists and breeders alike, in particular, for mapping newly obtained mutations, for selecting appropriate markers in accordance with breeding programs, and for studying pea genetic diversity using particular marker sequences.

## Supporting information

S1 Table CAPS markers used for *Sym13* genetic mapping. (DOCX)

S2 Table CAPS markers used for *Sym27* genetic mapping. (DOCX)

S3 Table List of *M*. *truncatula* genes located between *Medtr4g092760* and *Medtr4g096790*, including possible *Sym13* candidate genes. (XLSX)

S4 Table List of *M*. *truncatula* genes located between *Medtr7g058640* and *Medtr061018*, including possible *Sym27* candidate genes. (XLSX)
