# Supplementary material for: Pea Marker Database (PMD) – A new online database combining known pea (Pisum sativum L.) gene-based markers
Source: PLoS One. 2017 Oct 26;12(10):e0186713. doi: 10.1371/journal.pone.0186713 (PMC5658071; doi:10.1371/journal.pone.0186713)
Supplement: S1 Table — (DOCX) [file pone.0186713.s001.docx]

**Supplementary Table 1.** CAPS markers used for *Sym13* genetic mapping.

| Marker_PMD1 | Forward and reverse primers, 5’ - 3’ | Restriction enzyme |
| --- | --- | --- |
| PsC8268p528 | AACAAATAAAGATCGTGCCATCA | BclI |
|  | TCGAGGGCGGAGAGGATAAT |  |
| PsC5588p480 | GGGTGCCAACTGCAACAAAT | HpaII |
|  | ATGATTGGCCCCTCATCCTC |  |
| PsC908p622 | AGCGAATAGAGGAAACAGATCAA | HpaII |
|  | ACACGCCGAAAGAGTGGTAG |  |
| PsC28247p431 | GATACCTCAGCAGCCCAACC | Mph1103I |
|  | ACCTCACAGATGAGGGAAGC |  |
| Sen1 | GCGGTATTAGGAGCTAACGACGGATTAGTCT | BsmAI |
|  | TGAACCACAAAGCAAAGGAAC |  |
| Dnf2 | TATGGTGACATTTGGTTGTGTC | TaqI |
|  | GCCATCGGTTACCAGCATC |  |

Note that for *Sen1*, dCAPS marker was designed for distinguishing the nucleotide variant inherent in E135F line (*sym13*).
