# Supplementary material for: Pea Marker Database (PMD) – A new online database combining known pea (Pisum sativum L.) gene-based markers
Source: PLoS One. 2017 Oct 26;12(10):e0186713. doi: 10.1371/journal.pone.0186713 (PMC5658071; doi:10.1371/journal.pone.0186713)
Supplement: S2 Table — (DOCX) [file pone.0186713.s002.docx]

**Supplementary Table 2.** CAPS markers used for *Sym27* genetic mapping.

| Marker_PMD1 | Marker_PMD2 | Forward and reverse primers, 5’ - 3’ | Restriction enzyme |
| --- | --- | --- | --- |
| Ps001551 | Ps001551 | TCCAGTTTCTTCAACATAAACAACT | Eco91I |
|  |  | GAACCGTGGACGGAGCATTA |  |
| SNP_100000264 | PsCam051743_34202_196 | TGTTCCACAAGAAAGAACGTACA | Eco91I |
|  |  | TGGTTCAGTCGGAAAGCCAA |  |
| SNP_100000156 | - | TCAAAACTGTCCTTGGTAATGCT | TasI |
|  |  | GTCTCAAAGCTGTTGCGCTC |  |
| ArfB3 | ArfB3 | CCACAAGCCCAATATTTTCCTCTC | Hin1II |
|  |  | ATGCGTTGTCACTCCCTTGT |  |
| Met2 | Met2 | AACTGTGGTTGCGGTACTAGC | RsaI |
|  |  | TTATTCTATAACTCCAAAAGGGCG |  |
| Ps001440 | Ps001440 | ACCGGAAACTTCCCGGAAAT | TaqI |
|  |  | AATGATGACAATTATGATGGCAAG |  |
| Ps001772 | Ps001772 | CATGTCTTCCGATTTCACCTCAT | AluI |
|  |  | TGGAACGTCGTCCTGCCTGTA |  |
| Pme1 | - | GTTCAAAACTGTGGCTGA | AluI |
|  |  | GTGTTCTGGTTTGGGTCTTCTC |  |
| PsC19206p67 | PsC19206p67 | CGTGCGAACGCAATAAGGAG | HinfI |
|  |  | TCACTTTATTCTGTAACTGGCTAAC |  |
| PsC23878p158 | PsC23878p158 | CATTTGGCGGGAAGAGAGTATT | TaiI |
|  |  | TGCAGCCATGTATACAACTCCAT |  |
| Pgd | - | GTAGTCGCGTCTATGTTCAAC | HinfI |
|  |  | TCATGGATAGATCCCTCTGG |  |
